# Supplementary material for: Privacy-preserving data sharing via probabilistic modeling
Source: Patterns (N Y). 2021 Jun 7;2(7):100271. doi: 10.1016/j.patter.2021.100271 (PMC8276015; doi:10.1016/j.patter.2021.100271)
Supplement: Document S1. Table S1 [file mmc1.pdf]

Patterns, Volume *2*

Supplemental information

Privacy-preserving data sharing

via probabilistic modeling

Joonas Jälkö, Eemil Lagerspetz, Jari Haukka, Sasu Tarkoma, Antti Honkela, and Samuel Kaski

1 Additional experiment on ARD data

Table S1 shows that using 40 mixture components slightly improves the fit for both male and female cases when compared against both private and non-private results with 10 mixture components.

	Coefficient	Number of cases	Original coef.	$\epsilon = 2.0$	$\epsilon = 4.0$	$\epsilon = \infty$	$\epsilon = \infty, k = 40$
0	OAD only	254	0.657 ± 0.108	0.474 ± 0.209	0.591 ± 0.189	0.887 ± 0.149	0.7 ± 0.121
1	OAD+Insulin	12	0.873 ± 0.304	0.846 ± 0.44	1.074 ± 0.427	1.124 ± 0.366	1.12 ± 0.257
2	Insulin only	117	1.68 ± 0.135	1.085 ± 0.312	1.313 ± 0.293	1.521 ± 0.206	1.587 ± 0.153

	Coefficient	Number of cases	Original coef.	$\epsilon = 2.0$	$\epsilon = 4.0$	$\epsilon = \infty$	$\epsilon = \infty, k = 40$
0	OAD only	1052	0.435 ± 0.049	0.502 ± 0.152	0.538 ± 0.12	0.532 ± 0.089	0.523 ± 0.061
1	OAD+Insulin	66	0.582 ± 0.129	0.816 ± 0.282	0.858 ± 0.234	0.864 ± 0.17	0.757 ± 0.136
2	Insulin only	480	1.209 ± 0.063	1.188 ± 0.205	1.257 ± 0.138	1.262 ± 0.123	1.296 ± 0.082

Table S1: **ARD study**, **ABOVE** : Females, **BELOW** : Males. Increasing the number of mixture components improves the fit. DP and synthetic non-DP ($\epsilon = \infty$) results are average over 100 runs, error denoting the standard deviation. The error in original coefficients shows the standard error for the regression model.
