# Supplementary material for: Privacy-preserving data sharing via probabilistic modeling
Source: Patterns (N Y). 2021 Jun 7;2(7):100271. doi: 10.1016/j.patter.2021.100271 (PMC8276015; doi:10.1016/j.patter.2021.100271)
Supplement: Document S2. Article plus supplemental information [file mmc2.pdf]

Article

Patterns

Privacy-preserving data sharing via probabilistic modeling

Highlights

- Probabilistic modeling produces strongly anonymized synthetic data
- The usability of the synthetic data is conditional on the quality of the models
- Same statistical discoveries can be made from synthetic data as with original data

Authors

Joonas Jälkö, Eemil Lagerspetz, Jari Haukka, Sasu Tarkoma, Antti Honkela, Samuel Kaski

Correspondence

joonas.jalko@aalto.fi (J.J.), samuel.kaski@aalto.fi (S.K.)

In brief

Generating synthetic data using privacy-preserving models is a promising method for sharing sensitive data. This paper proposes to view synthetic data generation through probabilistic modeling, which allows the improvement of data generation by incorporating prior knowledge into the generative model. The proposed approach allows us to counteract reduction in quality, which results from the obfuscation required for privacy, and as a result produces high-quality synthetic data with strong privacy guarantees.

Jälkö et al., 2021, Patterns *2*, 100271
July 9, 2021 © 2021 The Authors.
https://doi.org/10.1016/j.patter.2021.100271

CellPress

CellPress
OPEN ACCESS

Article

Privacy-preserving data sharing via probabilistic modeling

Joonas Jälkö,[1,7,*] Eemil Lagerspetz,[2] Jari Haukka,[3] Sasu Tarkoma,[2] Antti Honkela,[2,5,6] and Samuel Kaski[1,4,5,6,*]
[1]Helsinki Institute for Information Technology (HIIT), Department of Computer Science, Aalto University, Espoo, 00076, Finland
[2]Helsinki Institute for Information Technology (HIIT), Department of Computer Science, University of Helsinki, Helsinki 00014, Finland
[3]Department of Public Health, University of Helsinki, Helsinki 00014, Finland
[4]Department of Computer Science, University of Manchester, Manchester M13 9PL, UK
[5]Senior author
[6]These authors contributed equally
[7]Lead contact
*Correspondence: joonas.jalko@aalto.fi (J.J.), samuel.kaski@aalto.fi (S.K.)
https://doi.org/10.1016/j.patter.2021.100271

THE BIGGER PICTURE Open data are a key component of open science. Unrestricted access to datasets would be necessary for the transparency and reproducibility that the scientific method requires. So far, openness has been at odds with privacy requirements, which has prohibited the opening up of sensitive data even after pseudonymization, which does not protect against privacy breaches using side information. A recent solution for the data-sharing problem is to release synthetic data drawn from privacy-preserving generative models. We propose to interpret privacy-preserving data sharing as a modeling task, allowing us to incorporate prior knowledge of the data-generation process into the generator model using modern probabilistic modeling methods. We demonstrate that this can significantly increase the utility of the generated data.

1 2 3 4 5 **Proof-of-Concept:** Data science output has been formulated, implemented, and tested for one domain/problem

SUMMARY

Differential privacy allows quantifying privacy loss resulting from accession of sensitive personal data. Repeated accesses to underlying data incur increasing loss. Releasing data as privacy-preserving synthetic data would avoid this limitation but would leave open the problem of designing what kind of synthetic data. We propose formulating the problem of private data release through probabilistic modeling. This approach transforms the problem of designing the synthetic data into choosing a model for the data, allowing also the inclusion of prior knowledge, which improves the quality of the synthetic data. We demonstrate empirically, in an epidemiological study, that statistical discoveries can be reliably reproduced from the synthetic data. We expect the method to have broad use in creating high-quality anonymized data twins of key datasets for research.

INTRODUCTION

The open release of data would be beneficial for research but is not feasible for sensitive data, for instance, clinical and genomic data. Since reliably anonymizing individual data entries is hard, releasing synthetic microdata[1] has been proposed as an alternative. To maximize the utility of the data, the distribution of the released synthetic data should be as close as possible to that of the original dataset, but should not contain synthetic examples that are too close to real individuals, as their privacy could be compromised. Traditional methods of statistical disclosure limitation cannot provide rigorous guarantees on the risk.[2] However, differential privacy (DP) provides a natural means of obtaining such guarantees.

DP[3,4] provides a statistical definition of privacy and anonymity. It gives strict controls on the risk that an individual can be identified from the result of an algorithm operating on personal data. Formally, a randomized algorithm $\mathcal{M}$ is (ε, δ)-DP, if for all datasets X, X', where X and X' agree in all but one entry, and for all possible outputs S of $\mathcal{M}$, it satisfies:

This is an open access article under the CC BY license (http://creativecommons.org/licenses/by/4.0/).

$$\Pr(\mathcal{M}(X) \in S) \leq e^{\varepsilon}\Pr(\mathcal{M}(X') \in S) + \delta, \quad \text{(Equation 1)}$$

where $0 \leq \delta < 1$. The non-negative parameters ε, δ define the strength of the guarantee, with smaller values indicating stronger guarantees. Privacy is usually achieved by introducing noise into the algorithms. DP has many desirable properties, such as composability: combining the results of several DP algorithms still produces DP, with privacy guarantees depending on how the algorithms are applied.[4,5] Another important property of DP is invariance to post-processing[6], which ensures that the privacy guarantees of a DP result remain valid after any post-processing. Thus we can use the results of a DP algorithm to answer future queries and still have the same privacy guarantees.

Data-sharing techniques under DP can be broadly separated into two categories as noted by Leoni[7]: input perturbation, where noise is added to the original data to mask individuals, and synthetic microdata, created from generative models learned under DP. The input perturbation techniques lack generality as they are often suitable for only very specific types of data, for example, set-valued data.[8] From now on we will focus only on synthetic-data-based techniques. Using DP for releasing synthetic microdata provides a more generalizable solution and was first suggested by Blum et al.[9] for binary datasets. Since then, multiple privacy-preserving data release techniques have been proposed.[10–16] However, the methods have so far been limited to special cases, such as discrete data[10,12–15,17] or having to draw a synthetic dataset from noisy histograms.[15,16] More recent work has employed more powerful models.[11,18,19] These methods have been shown to be much more efficient and general compared with previous attempts. However, these methods, as well as other data-sharing works, share a limitation: they are not able to use existing (prior) knowledge about the dataset.

Typically, the data-sharing methods are built around a similar idea: learn a generative model from the sensitive data under privacy guarantees and then sample a synthetic dataset from the trained model. These works differ mainly in the specific model used and how the model is learned under DP. Now one might ask, is this not sufficient, if the model is a universal approximator (such as variational autoencoders in Ács et al.[19]) and a sufficient amount of data are used to train it? The answer is yes, in principle, but in practice the amount of data required may be completely infeasible, as the universal approximator would need to learn from the data the structure of the problem, the causality, and all parameters. All this is made more difficult by the capacity of the models being more limited under DP and the necessary tuning of hyperparameters coming with a privacy cost.

If the human modeler has knowledge of how the data have been generated, it is much more data efficient to put this knowledge into the model structure than to learn everything from scratch with general-purpose data-driven models. For example, the data analyst might want to explicitly model structural zeros, i.e., zeros that correspond to an impossible outcome due to other features of the data, e.g., living subjects cannot have a cause of death. This is where the general purpose models fall short. Instead of building a new general purpose model for private data sharing, we propose a new essential component to private data sharing by augmenting the standard data-sharing workflow with a modeling task. In this modeling task, the user can encode existing knowledge of the problem and the data into the model before the private learning, thus guiding the DP learning task without actually accessing any private data yet.

We propose to give the modeler the tools of probabilistic modeling that provide a natural language to describe existing knowledge about how the data have been generated. This includes any prior knowledge, which can be seamlessly integrated. In a continuous or high-dimensional data space there is also another reason probabilistic modeling is needed: finite datasets are often sparse and require smoothing that preserves the important properties of the data.

In this paper we formulate the principle of "Bayesian DP data release," which employs a generative probabilistic model and hence turns synthetic data release into a modeling problem. We demonstrate how the modeling helps in data sharing by using a general purpose model as a starting point. We will increase the amount of prior knowledge encoded into the model and show empirically how the synthetic dataset becomes more similar to the original one when we guide it with more prior knowledge. We show how the modeling becomes pivotal in making correct statistical discoveries from the synthetic data. Code for applying the principle across model families and datasets is available at https://github.com/DPBayes/twinify (code for experiments in the paper is available at https://github.com/DPBayes/data-sharing-examples).

RESULTS

Overview of methods used in the experiments

Our aim is to release a new synthetic dataset that preserves the statistical properties of the original dataset while satisfying DP guarantees. Consider a dataset $\mathbf{X}$ and a probabilistic model $p(\mathbf{X}|\boldsymbol{\theta})$ with parameters $\boldsymbol{\theta}$. We use the posterior predictive distribution (PPD) $p(\tilde{\mathbf{X}}|\mathbf{X})$,

$$p\left(\tilde{\mathbf{X}}\middle|\mathbf{X}\right) = \int_{\mathrm{Supp}(\boldsymbol{\theta})} p\left(\tilde{\mathbf{X}}\middle|\boldsymbol{\theta}\right) p(\boldsymbol{\theta}|\mathbf{X})\,\mathrm{d}\boldsymbol{\theta}, \quad \text{(Equation 2)}$$

to generate the synthetic data. PPD tells us the probability of observing a new sample conditioned on the data we have obtained thus far. Therefore, if our model sufficiently captures the generative process, the PPD is the natural choice for generating the synthetic data. We sample the synthetic data from the PPD, by first drawing $\tilde{\boldsymbol{\theta}}$ from the posterior distribution $p(\boldsymbol{\theta}|\mathbf{X})$ and then drawing new data point $\tilde{\mathbf{x}}$ from the probabilistic model conditioned on $\tilde{\boldsymbol{\theta}}$, and repeating for all points.

Many of the previous differentially private data-sharing works share a common workflow, namely, they learn a specific generative model from the data and share samples drawn from this generator. This pipeline is depicted in Figure 1.

What we suggest is to augment this pipeline with domain knowledge of the data holder. This is possible through probabilistic modeling, which gives a natural language for encoding such knowledge prior to learning. In out experiments, we have used the new improved pipeline, depicted in Figure 2.

CellPress
OPEN ACCESS

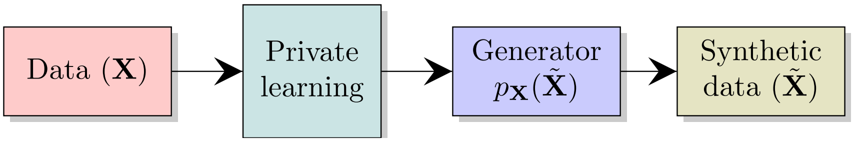

Figure 1. Standard differentially private data-sharing workflow

Reproducing statistical discoveries from the synthetic data

In order for private data sharing to be useful, we need to retain important statistical information in the synthetic data while preventing reidentification of data subjects. Next we will demonstrate how encoding prior knowledge becomes essential in making correct statistical discoveries from the synthetic data.

To test whether the same discoveries can be reproduced from the synthetic as from the original dataset, we generated a synthetic replica of a dataset used in an epidemiological study[20], using a general-purpose generative model family (mixture model). Prior to learning, we encoded experts' domain knowledge about the data into the probabilistic model.

The data have previously been used to study the association between diabetes and alcohol-related deaths (ARDs) using a Poisson regression model.[21] The study showed that males and females exhibit different behaviors in terms of alcohol-related mortalities. We encoded this prior knowledge into the model by learning independent mixture models for males and females. Another type of prior knowledge we had comes from the nature of the study that produced the data: the data of each subject end either on a specific date or at death. Hence, the status at the endpoint is known to have a one-to-one correspondence on certain features, such as duration of the follow-up and, most importantly, the binary indicator that tells if an individual died of alcohol-related causes. We encoded this prior knowledge into the probabilistic model as well. For details on the models we refer the reader to the experimental procedures.

After building the model, we learned the generative model under DP and generated the synthetic data. We fit the same Poisson regression model that was used in the earlier study[21] to the synthetic data as well, and compared the regression coefficients of the two models.

From the synthetic data, we make two key observations. (1) We can reproduce the discovery that diabetics have a higher risk of ARD than non-diabetics, which agrees with the previous results on the original data.[21] The bar dubbed "Stratified" in Figure 3 shows that we can reproduce the discoveries with high probability for males with relatively strict privacy guarantees ($\varepsilon = 1$). For females, we need to loosen the privacy guarantees to $\varepsilon = 4$ in order to reproduce the statistical discovery with high probability. We discuss the difference between males and females in the next section. (2) To reproduce the discovery, we need to have the correct model. Figure 3 shows the results of three different models: "Stratified," equipped with prior knowledge on gender and outcome of the follow-up; "No alive/dead strat.," with prior knowledge only on gender; and "Unstratified," without either type of prior knowledge. We see that the more prior knowledge we encode into the model, the better reproducibility we get. For males, with strict privacy ($\varepsilon = 1$) we increase the rate of reproducibility almost by 40% by having the correct model. For females, the effect is even stronger; however, it is best visible with larger ε.

Performance of DP data sharing

Next we will demonstrate the usability as well as the limitations of the proposed general DP data-sharing solution.

DP data sharing works best when data are plentiful

As we saw in Figure 3, the utility is better for males than the females, especially for strict privacy guarantees. To understand the difference between the two cases (males, females) in the ARD study, we note the much smaller sample size for ARD incidences among females (520 versus 2,312). Since DP guarantees indistinguishability among individuals in the dataset, it is plausible that the rarer a characteristic, the less well it can be preserved in DP-protected data. To assess whether this holds for the regression coefficients in the ARD study, we divided the regression coefficients, both male and female, into four equal-sized bins based on how many cases exhibited the corresponding feature and computed the mean absolute error between the original and the synthetic coefficients within these bins. Figure 4 shows that the regression coefficients with higher numbers of cases are more accurately discovered from the synthetic data.

Previously, Heikkilä et al.[22] showed that the error of estimating parameter mean under (ε, δ)-DP decreases proportional to $\mathcal{O}(1/n)$, where n is the size of the dataset. Figure 4 shows that the error in the ARD study follows closely the expected behavior as the number of cases increases. In this experiment, the inverse group size was estimated with the average of the inverse group sizes within a bin.

However, the data size is not the only determining factor for the utility of DP data sharing. Next we will show how more clear-cut characteristics of the data are easier to discover, even with fewer samples.

Picking up a weak statistical signal is difficult for DP data sharing

The ARD study stratifies individuals based on three types of diabetes treatment: insulin only, orally administered drug (OAD) only, and insulin + OAD treatment. Each of these therapies is treated as an independent regressor. For a reproduced discovery, we require that all of the regressors are positive and have sufficient statistical significance ($p < 0.05$). From Figure 5 we see that the probability of reproducing the discoveries for each subgroup increases as ε grows. However, we also see that for the insulin-only subgroup we recover the correct discovery with higher rate compared with the larger subgroup, OAD only. The reason the smaller subgroup of insulin only is captured with sufficient significance more often than the largest subgroup, OAD only, can be explained by the original regression coefficients shown in Table 1. The OAD-only subgroup has a significantly smaller effect on the ARD than the insulin-only subgroup, thus making it more difficult for the mixture model to capture. However, as we increase ε, the correlation between OAD only and ARD is more often captured. Both of these effects are also visible in the male case, as we see from Figure 5, but on a smaller scale.

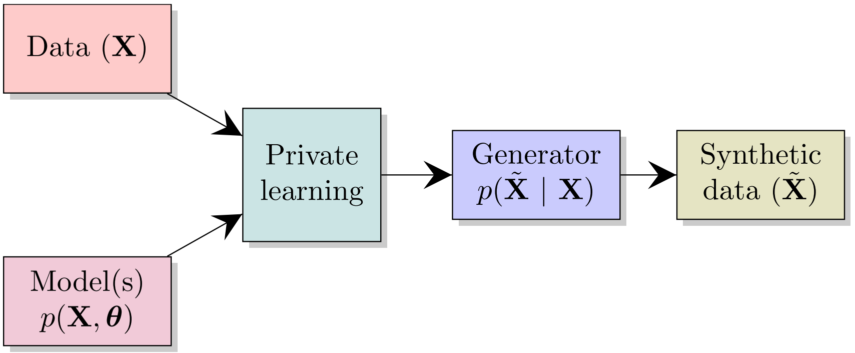

Figure 2. Bayesian DP data release

with Gaussian noise, according to the Gaussian mechanism[3] ("tailored mechanism"). We measured the accuracy in terms of the Frobenius norm (see Equation 8) between the true and the DP covariance matrices. Already with $T = 10$ queries, releasing a synthetic dataset outperforms the tailored mechanism for these high-dimensional data. We show results only for the mixture model because the difference in performance between the mixture model and the Bayes networks is small in this example (see Figure 8).

As another example, we compared the synthetic data release on the Adult data against a private logistic regression classifier.[26] Figure 7 shows that the Bayes network consistently outperforms the tailored mechanism, and for strict privacy requirement (small ε) the mixture model also performs better than the tailored mechanism, given 20 or more queries.

Some of the regression coefficients learned from the synthetic data diverge from the ground truth, which seems to also persist without privacy (see column $\varepsilon = \infty$ in Table 1). In our experiments we have used a small number of mixture components ($k = 10$) as a compromise between sufficiently high resolution (we can make correct statistical discoveries) and private learning that becomes more difficult as the number of parameters grows. Increasing the number of mixture components resolves this inconsistency by improving the fit in the non-private case (see Table S1 in the supplemental information).

To evaluate the strength of the statistical signals in the female ARD study, we ran the Poisson regression study with bootstrapped original female data. Figure 6 shows that under 100 bootstrap iterations, ≈30% of the repeats did not reach the required statistical significance. This shows that the statistical signal in female data is weak to begin with, and therefore may be difficult for a data-sharing model to capture.

Despite DP data sharing having difficulties with weak statistical signal and limited data, it provides an efficient solution for privacy-preserving learning, especially when we are not certain about the future use of the data. Next we will discuss how DP-based synthetic data stand against traditional query-based DP approaches.

Performance against a tailored mechanism

As discussed, one of the greatest advantages of releasing a synthetic dataset is that it can be used in arbitrary tasks without further privacy concerns. Using traditional DP techniques, a data holder that wants to allow DP access to a sensitive dataset needs to set a privacy budget at the desired level of privacy and split this budget for each access that the data are subjected to. As soon as the privacy budget runs out, the data cannot be used in any additional analysis without unacceptable privacy risk.

We will next show that the data-sharing methods can outperform traditional DP techniques, if the data are to be accessed multiple times. We evaluate the performance on two datasets, a mobile phone app dataset[23] referred to as Carat and the publicly available set of US census data, "Adult".[24] As data-sharing methods we apply a mixture-model-based PPD sampling method ("mixture model") and a Bayes-networks-based method, PrivBayes[25] ("Bayes network").

Consider that the data holder splits the budget uniformly among T anticipated queries. Figure 7 illustrates how the number of anticipated queries will affect the accuracy. We compared the data-sharing method against perturbing the covariance matrix

Demonstration on two parametric families of distributions

Finally, we will demonstrate the results from two data-sharing approaches using two very different universal probabilistic models making different computational trade-offs. We evaluate the performance between mixture models and Bayes networks on the ARD, Carat, and Adult datasets.

For the Carat data, Figure 8, left, shows that the Bayes network is accurate when the dimensionality of the data is low, but as the dimensionality grows, synthetic data generated from the mixture model achieve higher accuracy than data from Bayes networks, which also become computationally exhausting as the dimension increases. From Figure 8, we can see that learning the mixture model takes only a fraction of the Bayes networks' computational time. Similarly, in the ARD study, the mixture models outperforms Bayes networks (Figure 8, right).

As a final comparison between the Bayes networks and the mixture model, we compared the two in the previously introduced classification task using the Adult dataset, which has fewer samples compared with ARD and Carat data (Adult 30,162 samples, Carat 66,754 samples, ARD females 208,148 samples, and ARD males 226,372). After the generative model was learned, we used the synthetic data obtained from the generative model to train a logistic regression classifier and demonstrated the performance by predicting income classes. Figure 7, right, illustrates that in this example, the Bayes networks outperform the mixture model in terms of classification accuracy.

DISCUSSION

Dwork et al.[27] showed theoretically that there is no computationally efficient DP method for data sharing that would preserve all properties of the data. They consider the problem from the learning theory perspective, where the aim is to accurately answer a set of queries. Accurate answers become infeasible as the size of this query set grows. However, if we need only to

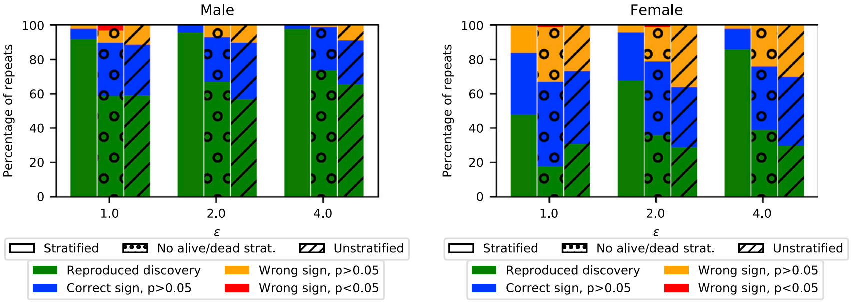

Figure 3. ARD study: Encoding prior knowledge into the generative model improves performance

For both males (left) and females (right), we recover the correct statistical discovery with high probability when we guide the model sufficiently with prior knowledge. The prior knowledge is increased from right to left in both groups. In “Stratified,” we have independent mixture models for the genders and deterministic features due to study outcomes. In “No alive/dead strat.” we have independent models for the genders, and in “Unstratified” we treat all features within a mixture component as independent. For a reproduced discovery, we required the association between ARD and medication type to be found for all medication types with significance ($p < 0.05$). The results of 100 independent repeats of each method with three levels of privacy (parametrized by ε) are shown.

preserve the most important properties of the data, the set of queries we want to accurately answer stays bounded in size, giving a way out. We argue that it would already be highly useful to be able to answer questions of the important properties; and moreover, the bigger picture may be more relevant than all the unique characteristics in the data.

As we saw in the Adult example, the DP data release can perform as well as the tailored mechanism, even when answering just one query, and progressively better for multiple queries. However, as our experiments exemplify, encoding of the prior knowledge has a significant impact on the results. In fact, what we are proposing is to transform the DP data release problem into a modeling problem, which includes as an essential part the selection of the model according to the data and task, and bringing in available prior knowledge.

We illustrated in Figure 4 how increasing the number of relevant samples improves the results. As is common with all differentially private methods, the data release works better when the original dataset has a large number of samples. This is because of the nature of DP; it is easier to mask the contribution of one element of the dataset when the number of samples is large.

Recently, Karwa et al.[28] showed that DP has a broadening effect on the confidence intervals of statistical quantities learned under DP. Their proof was for Gaussian mean estimation; however, intuitively, this property should translate to other differentially private tasks as well. The width of the confidence intervals depends on both the required level of privacy and the number of samples. This suggests that we should not expect to necessarily reproduce all the same discoveries under DP.

In the past, there has been discussion on whether standard random number generators (RNGs) can be used to ensure DP.[29] In the actual data release setting we would need to consider using cryptographically secure RNGs to properly provide individuals in the dataset the DP guarantees. Also, the limited accuracy of floating point arithmetics makes it possible for an attacker to break DP due to errors in approximation.[30] However, these problems are by no means specific to DP data release but apply to all DP methods.

One major question for all DP algorithms is how to set the privacy parameters ε and δ. While the parameters are in principle well defined, their interpretation depends, for example, on the chosen neighborhood relation. Furthermore, the parameters are worst-case bounds that do not fully capture, for example, the fact that we do not release the full generative model but only samples drawn from the PPD. Our use of $\varepsilon \approx 1$ is in line with widely accepted standards derived from observed feasibility of membership inference attacks. Given the complicated relationship between the released and the original data, it seems unlikely that the privacy of specific data subjects could be compromised in this setting under this privacy level.

In this work, we have reformulated the standard differentially private data sharing by formulating it as a modeling task. Using probabilistic modeling, we can express prior knowledge about the data and processes that generated the data before the training, thus guiding the model toward the right directions without additional privacy cost. This makes it possible to extend the DP data sharing solution to datasets that are of limited size, but for which there exists domain knowledge.

Differentially private data sharing shows great potential, and would be particularly useful for datasets that will be used in multiple analyses. Census data are a great example of such data. Also, as private data sharing allows arbitrary downstream

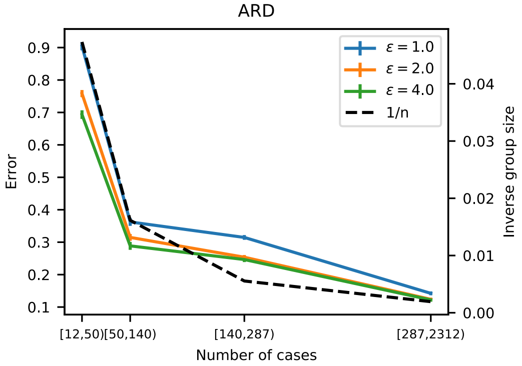

Figure 4. Accuracy of findings from synthetic data as a function of their rarity: ARD study

The accuracy of regression coefficients learned from synthetic data rapidly improves as the number of relevant samples grows. The solid curves show mean absolute error within a prevalence bin between the regression coefficients learned from original and synthetic data. The average result over 100 independent runs of the algorithm is shown. The dashed line is proportional to the expected behavior of an optimal estimator (see text); note the different scale on the y axis (shown on the right). Results are from the stratified model. Tick marks on the x axis are (min, max) number of relevant samples within the respective bin. Error bars denote the standard error of the mean. Results shown are for three values of the privacy parameter ε.

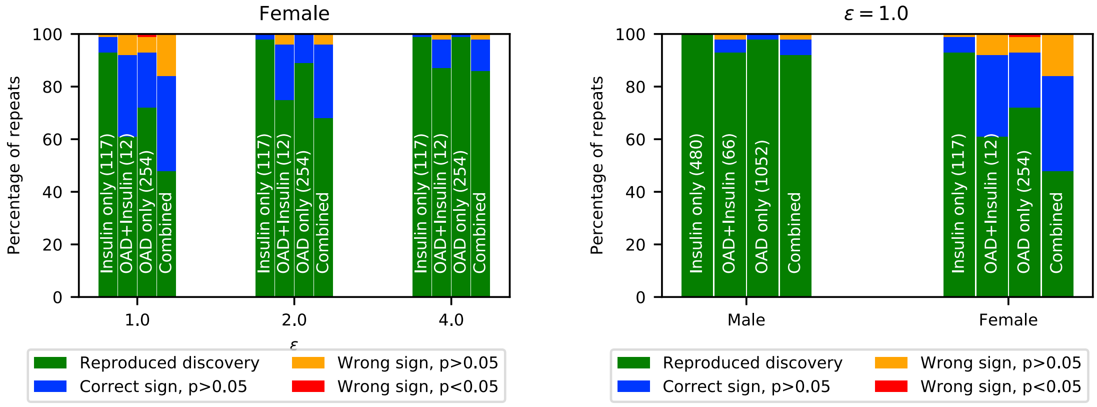

Figure 5. The statistical signal is weaker in female data (ARD study)

(Left) Likelihood of reproducing findings as a function of privacy guarantee, female case. The statistical discoveries are reliably reproduced from the synthetic data for the strictest privacy requirements. Results are for the combined case and each subgroup separately. In the combined results, all subgroups are required to have the correct sign and $p < 0.05$ to call the discovery reproduced. The size of each subgroup is shown in parentheses. Results are from the stratified model. (Right) Likelihood of reproducing findings from synthetic data. For males (226,372 samples), the discoveries can be reproduced with high probability from the synthetic data. For females (208,148 samples), the probability of reproducing discoveries is lower. Bars show discoveries for each type of diabetes medication separately and for all combined. In the combined case, for a reproduced discovery, we required the association between ARD and medication type to be found for all medication types with significance ($p < 0.05$). The results of 100 independent repeats of the method with privacy level ($\varepsilon = 1.0, \delta = 10^{-6}$) using the stratified model are shown.

tasks with no further privacy cost, it is a good alternative for tasks for which there is no existing privacy-preserving counterpart.

Our results demonstrate the importance of guiding the data-sharing task with prior knowledge about the data domain, and that when this prior knowledge is encoded into the probabilistic model, the synthetic data maintain the usability of the original data in non-trivial tasks.

EXPERIMENTAL PROCEDURES

Resource availability

Lead contact

Joonas Jälkö is the lead contact for this work and can be contacted by email at joonas.jalko@aalto.fi.

Materials availability

Synthetic datasets generated for the publicly available Adult dataset can be requested from the authors.

Data and code availability

The code used in our experiments is available at https://github.com/DPBayes/data-sharing-examples.

The Adult dataset is available from the UCI machine learning repository (https://archive.ics.uci.edu/ml/datasets/adult).

The ARD and Carat datasets contain personal information and therefore are not publicly available.

Regarding the Carat data gathering process, the user was informed about the data gathering and the research usage of the data (including app data) when installing the application in the End-User License Agreement. The process complies with EU's General Data Protection Regulation. The app requires user consent for the installation. The developers have IRB (ethical board) approval for the Carat data gathering and analysis. An anonymized subset of Carat data can be found at https://www.cs.helsinki.fi/group/carat/data-sharing/.

The ARD data were a collection from multiple sources: Social Insurance Institute (SII; permission Kela 16/522/2012), the Finnish Cancer Registry, National Institute for Health and Welfare (THL/264/5.05.00/2012), and Statistics Finland (TK-53-214-12). This is a register-based study with pseudonymous data and no patient contact, thus no consents from pseudonymized patients were required according to Finnish law. The ethical committee of the Faculty of Medicine, University of Helsinki, Finland (02/2012) reviewed the protocol. Data permits were received from the SII (16/522/2012), the National Institute for Health and Welfare (THL/264/5.05.00/2012), and Statistics Finland (TK-53-214-12). The SII pseudonymized the data.

Table 1. ARD study

Coefficient	Number of cases	Original coefficient ± SE	ε = 1.0	ε = 2.0	ε = 4.0	ε = ∞
Females						
OAD only	254	0.657 ± 0.108	0.303 ± 0.197	0.474 ± 0.209	0.591 ± 0.189	0.887 ± 0.149
OAD + insulin	12	0.873 ± 0.304	0.658 ± 0.516	0.846 ± 0.44	1.074 ± 0.427	1.124 ± 0.366
Insulin only	117	1.68 ± 0.135	0.91 ± 0.379	1.085 ± 0.312	1.313 ± 0.293	1.521 ± 0.206
Males						
OAD only	1,052	0.435 ± 0.049	0.412 ± 0.166	0.502 ± 0.152	0.538 ± 0.12	0.532 ± 0.089
OAD + insulin	66	0.582 ± 0.129	0.748 ± 0.304	0.816 ± 0.282	0.858 ± 0.234	0.864 ± 0.17
Insulin only	480	1.209 ± 0.063	1.033 ± 0.189	1.188 ± 0.205	1.257 ± 0.138	1.262 ± 0.123

The magnitude of the statistical effect in the male case is well preserved in synthetic data. DP and synthetic non-DP ($\varepsilon = \infty$) results are averaged over 100 runs, with error denoting the standard deviation. The error in the original coefficients shows the standard error for the regression model.

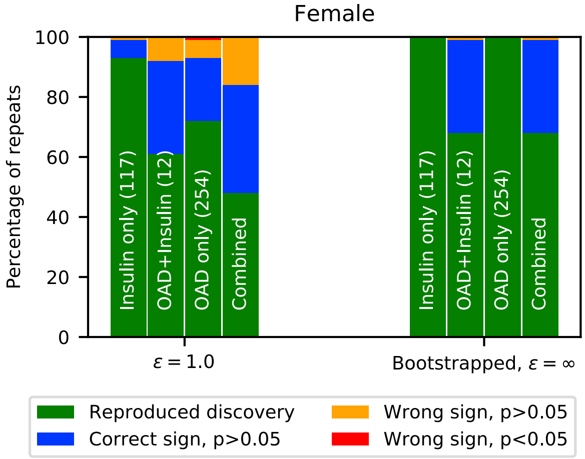

Figure 6. ARD study

The statistical signal is weak in the female data, and discoveries cannot be made with sufficient significance. On the left, the bars show results for private synthetic data with $\varepsilon = 1.0$ of 100 independent runs using the stratified model. On the right, the bars show results for 100 times bootstrapped original data.

Materials

For the ARD study[20], the data came from 208,148 females and 226,372 males and comprised three continuous, five binary, and two categorical features.

Carat[23] is a research project that maintains a mobile phone app that helps users understand their battery usage. For the Carat dataset, we obtained a subset of Carat data from the research project. Our aim was to privately release a dataset that consists of installed apps of 66,754 Carat users. To have some variance in the data, we dropped out the 100 most popular apps that were installed on almost every device and used the 96 next most popular apps to subsample in the experiments.

In the Adult study of the UCI machine learning repository,[24] we trained the generative model with 30,162 samples with 13 features of both continuous and discrete types. A separate test set consisted of 15,060 instances, of which 75.4% were labeled $\leq$50k$.

Differential privacy

In our experiments, we used approximate DP, as defined below.

Definition 1 (approximate DP[3]): a randomized algorithm $\mathcal{M} : \mathcal{X}^N \rightarrow \mathcal{I}$ satisfies (ε, δ) DP, if for all adjacent datasets $X, X' \in \mathcal{X}^N$ and for all measurable $S \subset \mathcal{I}$ it holds that:

$$\Pr(\mathcal{M}(X) \in S) \leq e^{\varepsilon} \Pr(\mathcal{M}(X') \in S) + \delta. \quad \text{(Equation 3)}$$

We consider datasets as adjacent in the substitute relation, i.e., if we get one by replacing a single element of the other and vice versa. The privacy parameter δ used in the experiments was set to 10^{-6} for the ARD study and 10^{-5} for both the Carat and the Adult studies.

Probabilistic models

Mixture model

Mixture model is a universal approximator of densities. The probability density for a mixture model with K mixture components is given as:

$$p\left(\mathbf{X}|\boldsymbol{\theta}, \boldsymbol{\pi}\right) = \sum_{k=1}^{K} \boldsymbol{\pi}_k p\left(\mathbf{X}|\boldsymbol{\theta}^{(k)}\right). \quad \text{(Equation 4)}$$

It allows the capture of complex dependency structures through the differences between less complex mixture components (the densities $p(\mathbf{X}|\boldsymbol{\theta}^{(k)})$). There is no limitation on what kinds of distributions can be used for the mixture components, and thus a mixture model is suitable for arbitrary types of data. In this work we assume independence of features within each mixture component. This means that the component distribution factorizes over the features, and we can write:

$$p\left(\mathbf{X}|\boldsymbol{\theta}, \boldsymbol{\pi}\right) = \sum_{k=1}^{K} \boldsymbol{\pi}_k \prod_{j=1}^{D} p\left(\mathbf{X}_j|\boldsymbol{\theta}_j^{(k)}\right), \quad \text{(Equation 5)}$$

where $\mathbf{X}_j, j = 1, \ldots, D$ denotes the D features of the data and $\boldsymbol{\theta}_j^{(k)}$ the parameters associated with the jth feature of the kth component distribution. Intuitively the problem can be seen as finding clusters of features such that each cluster has an axis-aligned covariance structure. As the number of such clusters increases, we can cover the data more accurately.

In our experiments with mixture models, we used PPD as the generative model. The only access to data is through the posteriors of the model parameters, which we learned under DP using the differentially private variational inference (DPVI) method.[26] DPVI learns a mean field approximation for the posterior distributions of model parameters using DP-SGD.[31] The number of mixture components K was set to 10 for data with fewer dimensions (<20) and to 20 for data with more dimensions ($\geq$20). If necessary, this number, along with hyperparameters of DPVI, could be optimized under DP[32], with potentially significant extra computational cost.

Bayes networks

A Bayes network is a graphical model that presents the dependencies across random variables as a directed acyclic graph. In the graph, the nodes represent random variables and the edges dependencies between the variables. To learn the graphs privately and to sample the synthetic data, we used the PrivBayes method,[25] which builds the graph between the features of the data, and no additional latent variables were assumed. The topology of the network is chosen under DP by using the exponential mechanism,[33] and the conditional distributions that describe the probability mass function are released using the Laplace mechanism.[4]

Model details

For the mixture model, we need to choose how to model each feature in the datasets. In all our experiments we used the following distributions: continuous features were scaled to the unit interval and modeled as beta distributed.

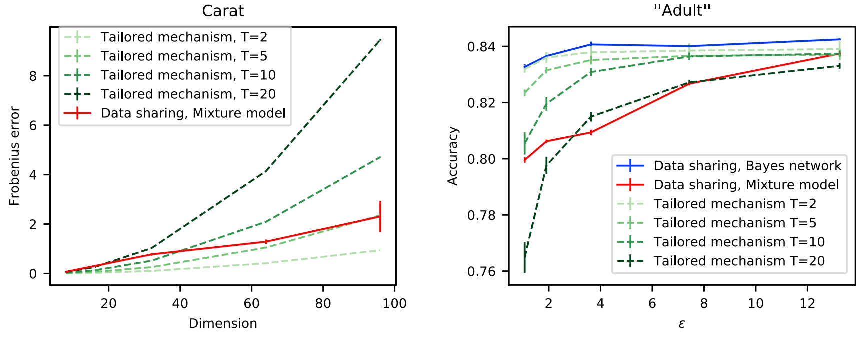

Figure 7. Performance against tailored mechanisms

(Left) Carat study. The data-sharing method outperforms the tailored mechanism as the number of anticipated future queries (T) grows, in terms of classification accuracy. Curves show the Frobenius norm between original and synthetic covariance matrices. Privacy budget was fixed to $(1.0, 10^{-5})$. The average of 10 runs is shown. Error bars denote the standard error of mean. (Right) Adult study. Synthetic data from the Bayes network model outperform the tailored mechanism. While a tailored mechanism is more accurate for loose privacy guarantees (large ε) and few queries (small T), the mixture-model-based data release is more accurate for multiple queries and tighter privacy guarantees. The average classification accuracy over 10 independent runs is shown. Error bars denote standard error of mean.

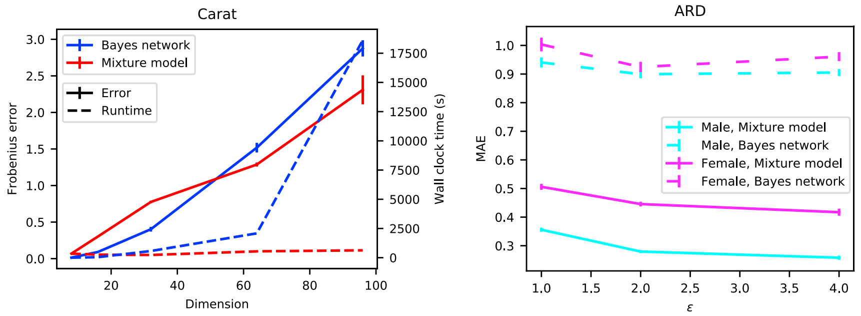

Figure 8. Comparing mixture model and Bayes networks using two different datasets

(Left) Accuracy and computation speed of two models in generating synthetic data (Carat study). For low-dimensional discrete data, Bayes networks are good, but as dimensionality grows, their computation time becomes intolerable and mixture models become more accurate. The solid lines denote the mean Frobenius norm (see Equation 8) between the original and the synthetic covariance matrices, with error bars denoting standard error of the mean from 10 independent runs of the algorithm. The dashed lines show the run times. Privacy budget was fixed to $(\varepsilon = 1.0, \delta = 10^{-5})$. (Right) Accuracy of data synthesized with two models (ARD study). Mixture models preserve regression coefficients better than the Bayes network. The curves show mean absolute error between the original and the learned coefficients. The average over 100 runs is shown. Error bars indicate the standard error of the mean.

The parameters for beta-distributed variables were given a gamma$(1,1)$ prior. Discrete features were modeled as either Bernoulli or categorical random variables based on the domain. In both Bernoulli and categorical cases, the parameters were given a uniform prior. Table 2 summarizes the mixture models used in the experiments.

Prior knowledge used in the ARD study

In the ARD study, we showed how incorporating prior knowledge into the model improves the utility of data sharing. Next we will describe in detail the type of knowledge we used to model the data. We will encode the prior knowledge into the mixture model given in Equation (5). This corresponds to the model referred to as "Unstratified" in Figure 3.

We start by splitting the probabilistic model based on gender of the subject. This yields the following likelihood function:

$$p(\mathbf{X}|\boldsymbol{\theta},\boldsymbol{\pi}) = p(x_{\text{sex}}|\theta_{\text{sex}})\sum_{k=1}^{K}\boldsymbol{\pi}_k p\left(\mathbf{X}_{\setminus\{\text{sex}\}}\middle|\boldsymbol{\theta}^{(k)}, x_{\text{sex}}\right). \quad \text{(Equation 6)}$$

We refer to this model as "No alive/dead strat."

The ARD data are an aggregate of a follow-up study, which ended either on December 31, 2012, or on the subject's death. In this study, we were interested in whether an individual died due to alcohol-related reasons. Since the subject cannot be dead due to alcohol-related reasons while still continuing to the end of the follow-up, we separated the model according to subjects' status by the end of the follow-up. This led to the final "Stratified" model used in our experiments, with likelihood given as:

$$p(\mathbf{X}|\boldsymbol{\theta},\boldsymbol{\pi}) = p(x_{\text{sex}}|\theta_{\text{sex}})p(x_{\text{dead}}|x_{\text{sex}},\theta_{\text{dead}}) \sum_{k=1}^{K}\boldsymbol{\pi}_k p\left(\mathbf{X}_{\setminus\{\text{dead, sex}\}}\middle|\boldsymbol{\theta}^{(k)}, x_{\text{dead}}, x_{\text{sex}}\right). \quad \text{(Equation 7)}$$

Here, x_{dead} denotes the end-of-follow-up indicator and $\mathbf{X}_{\setminus\{\text{dead}\}}$ the features of the data excluding the end-of-follow-up indicator. Now we could learn two mixture models, one for living and the other for dead subjects, for both females and males. Since the living subjects stayed in the study until the end of the follow-up, we could model the feature pair ("start date" and "duration of follow-up") using just one of the features. In our experiments we used the "start date" feature. Similar to the ARD, as death could occur only in dead subjects, we could remove this feature from the living model.

Table 2. Summary of mixture model details

Dataset	K	Variable types	Details
ARD	10	binary, categorical, beta	separate mixture models for males and females and separation based on outcome of the follow-up
Carat	20	binary	within a mixture component, the features were treated as independent
Adult	10	binary, categorical, beta	separate mixture models for high/low income; "hours per week," "capital loss," and "capital gain" features were discretized into 16 bins

Similarity measures

In the Carat experiments, we measured the performance in terms of the similarity between the covariance matrices of the original and the synthetic data. The Frobenius norm between two matrices, A and B, is given as:

$$||A-B||_F = \left(\sum_{i=1}^{n}\sum_{j=1}^{d}(a_{ij}-b_{ij})^2\right)^{1/2}. \quad \text{(Equation 8)}$$

SUPPLEMENTAL INFORMATION

Supplemental information can be found online at https://doi.org/10.1016/j.patter.2021.100271.

ACKNOWLEDGMENTS

This work was supported by the Academy of Finland (grants 325573, 325572, 319264, 313124, 303816, 303815, 297741, and 292334 and the Flagship program Finnish Center for Artificial Intelligence [FCAI]). We thank the Carat group for access to the Carat data (http://carat.cs.helsinki.fi/) and the CARING study group (https://www.caring-diabetes.eu/) for access to the ARD data.

AUTHOR CONTRIBUTIONS

J.J., S.K., and A.H. designed the research; J.J., E.L., and J.H. analyzed the data; J.J., E.L., J.H., S.T., S.K., and A.H. interpreted the results; and J.J., S.K., and A.H. wrote the paper.

DECLARATION OF INTERESTS

The authors declare no competing interests.

Received: January 1, 2021
Revised: February 27, 2021
Accepted: April 30, 2021
Published: June 7, 2021

REFERENCES

1. Rubin, D.B. (1993). Discussion: statistical disclosure limitation. J. Off. Stat. 9, 461–468.
2. Abowd, J.M., and Vilhuber, L. (2008). How protective are synthetic data? In Proc. of the PSD'08, volume 5262 of LNCS, J. Domingo-Ferrer and Y. Saygin, eds. (Springer), pp. 239–246.

3. Dwork, C., Kenthapadi, K., McSherry, F., Mironov, I., and Naor, M. (2006). Our data, ourselves: privacy via distributed noise generation. In EUROCRYPT 2006, volume 4004 of LNCS, S. Vaudenay, ed. (Springer), pp. 486–503.
4. Dwork, C., McSherry, F., Nissim, K., and Smith, A. (2006). Calibrating noise to sensitivity in private data analysis. In Theory of Cryptography. TCC 2006. Lecture Notes in Computer Science, *3876*, S. Halevi and T. Rabin, eds. (Springer). https://doi.org/10.1007/11681878_14.
5. Dwork, C., Rothblum, G.N., and Vadhan, S.P. (2010). Boosting and differential privacy. In FOCS 2010 (IEEE Computer Society), pp. 51–60. https://doi.org/10.1109/FOCS.2010.12.
6. Dwork, C., and Roth, A. (2014). The algorithmic foundations of differential privacy. Found. Trends Theor. Comput. Sci. *9*, 211–407. https://doi.org/10.1561/0400000042.
7. Leoni, D. (2012). Non-interactive differential privacy: a survey. In WOD '12, G. Raschia and M. Theobald, eds. (ACM), pp. 40–52. https://doi.org/10.1145/2422604.2422611.
8. Chen, R., Mohammed, N., Fung, B.C.M., Desai, B.C., and Xiong, L. (2011). Publishing set-valued data via differential privacy. Proc. VLDB Endow. *4*, 1087–1098.
9. Blum, A., Ligett, K., and Roth, A. (2008). A learning theory approach to non-interactive database privacy. In Proc. of STOC '08 (ACM), pp. 609–618. https://doi.org/10.1145/1374376.1374464.
10. Beimel, A., Kasiviswanathan, S.P., and Nissim, K. (2010). Bounds on the sample complexity for private learning and private data release. In Proc. of the TCC'10, Volume 5978 of LNCS, D. Micciancio, ed. (Springer), pp. 437–454. https://doi.org/10.1007/978-3-642-11799-2_26.
11. Chanyaswad, T., Liu, C., and Mittal, P. (2019). Ron-gauss: Enhancing utility in non-interactive private data release. Proc. Priv. Enh. Technol. *2019*, 26–46. https://doi.org/10.2478/popets-2019-0003.
12. Chen, R., Ács, G., and Castelluccia, C. (2012). Differentially private sequential data publication via variable-length n-grams. In Proc. of the CCS'12, T. Yu, G. Danezis, and V.D. Gligor, eds. (ACM), pp. 638–649. https://doi.org/10.1145/2382196.2382263.
13. Hardt, M., Ligett, K., and McSherry, F. (2012). A simple and practical algorithm for differentially private data release. In Proc. of NIPS'12, P.L. Bartlett, F.C.N. Pereira, C.J.C. Burges, L. Bottou, and K.Q. Weinberger, eds., pp. 2348–2356.
14. Mohammed, N., Chen, R., Fung, B.C., and Yu, P.S. (2011). Differentially private data release for data mining. In Proc. of the KDD'11 (ACM), pp. 493–501. https://doi.org/10.1145/2020408.2020487.
15. Xiao, Y., Xiong, L., and Yuan, C. (2010). Differentially private data release through multidimensional partitioning. In Proc. SDM'10, Volume 6358 of LNCS, W. Jonker and M. Petkovic, eds. (Springer), pp. 150–168. https://doi.org/10.1007/978-3-642-15546-8‘‘11.
16. Xiao, Y., Gardner, J.J., and Xiong, L. (2012). Dpcube: releasing differentially private data cubes for health information. In Proc. of the ICDE'12, A. Kementsietsidis and M.A.V. Salles, eds. (IEEE Computer Society), pp. 1305–1308. https://doi.org/10.1109/ICDE.2012.135.
17. Gupta, A., Roth, A., and Ullman, J.R. (2012). Iterative constructions and private data release. In Proc. of the TCC'12, Volume 7194 of LNCS, R. Cramer, ed. (Springer), pp. 339–356. https://doi.org/10.1007/978-3-642-28914-9_19.
18. Abay, N.C., Zhou, Y., Kantarcioglu, M., Thuraisingham, B.M., and Sweeney, L. (2018). Privacy preserving synthetic data release using deep learning. In Proc. of the ECML PKDD'18, Volume 11051 of LNCS, M. Berlingerio, F. Bonchi, T. Gärtner, N. Hurley, and G. Ifrim, eds. (Springer), pp. 510–526. https://doi.org/10.1007/978-3-030-10925-7_31.
19. Ács, G., Melis, L., Castelluccia, C., and Cristofaro, E.D. (2019). Differentially private mixture of generative neural networks. IEEE Trans. Knowl. Data Eng. *31*, 1109–1121. https://doi.org/10.1109/TKDE.2018.2855136.
20. But, A., De Bruin, M.L., Bazelier, M.T., Hjellvik, V., Andersen, M., Auvinen, A., Starup-Linde, J., Schmidt, M.K., Furu, K., de Vries, F., et al. (2017). Cancer risk among insulin users: comparing analogues with human insulin in the CARING five-country cohort study. Diabetologia *60*, 1691–1703. https://doi.org/10.1007/s00125-017-4312-5.
21. Niskanen, L., Partonen, T., Auvinen, A., and Haukka, J. (2018). Excess mortality in Finnish diabetic subjects due to alcohol, accidents and suicide: a nationwide study. Eur. J. Endocrinol. *179*, 299–306. https://doi.org/10.1530/EJE-18-0351.
22. Heikkilä, M.A., Lagerspetz, E., Kaski, S., Shimizu, K., Tarkoma, S., and Honkela, A. (2017). Differentially private bayesian learning on distributed data. In Proc. of the NIPS'17, I. Guyon, U. von Luxburg, S. Bengio, H.M. Wallach, R. Fergus, S.V.N. Vishwanathan, and R. Garnett, eds., pp. 3226–3235.
23. Oliner, A.J., Iyer, A.P., Stoica, I., Lagerspetz, E., and Tarkoma, S. (2013). Carat: collaborative energy diagnosis for mobile devices. In Proc. of the SenSys'13, C. Petrioli, L.P. Cox, and K. Whitehouse, eds. (ACM), pp. 10:1–10:14. https://doi.org/10.1145/2517351.2517354.
24. Dua, D., and Graff, C. (2019). UCI Machine Learning Repository (Irvine, CA: University of California, School of Information and Computer Science). http://archive.ics.uci.edu/ml.
25. Zhang, J., Cormode, G., Procopiuc, C.M., Srivastava, D., and Xiao, X. (2017). Privbayes: private data release via bayesian networks. ACM Trans. Database Syst. *42*, 4. https://doi.org/10.1145/3134428.
26. Jälkö, J., Dikmen, O., and Honkela, A. (2017). Differentially private variational inference for non-conjugate models. In Proc. of the UAI 2017, G. Elidan and K. Kersting, eds. (Association for Uncertainty in Artificial Intelligence), p. 152.
27. Dwork, C., Naor, M., Reingold, O., Rothblum, G.N., and Vadhan, S. (2009). On the complexity of differentially private data release: efficient algorithms and hardness results. In Proc. of the STOC '09 (ACM), pp. 381–390. https://doi.org/10.1145/1536414.1536467.
28. Karwa, V., and Vadhan, S.P. (2018). Finite sample differentially private confidence intervals. In Proc. of the ITCS'18, Volume 94 of LIPIcs, A.R. Karlin, ed. (Schloss Dagstuhl - Leibniz-Zentrum für Informatik), pp. 44:1–44:9. https://doi.org/10.4230/LIPIcs.ITCS.2018.44.
29. Garfinkel, S.L., and Leclerc, P. (2020). Randomness concerns when deploying differential privacy. In Proc. of the WPES'20 (Association for Computing Machinery), pp. 73–86. https://doi.org/10.1145/3411497.3420211.
30. Mironov, I. (2012). On significance of the least significant bits for differential privacy. In Proc. of the CCS'12 (ACM), pp. 650–661. https://doi.org/10.1145/2382196.2382264.
31. Abadi, M., Chu, A., Goodfellow, I., McMahan, H.B., Mironov, I., Talwar, K., and Zhang, L. (2016). Deep learning with differential privacy. In Proc. of the CCS'16 (ACM), pp. 308–318. https://doi.org/10.1145/2976749.2978318.
32. Liu, J., and Talwar, K. (2019). Private selection from private candidates. In Proc. of the STOC'19 (ACM), pp. 298–309. https://doi.org/10.1145/3313276.3316377.
33. McSherry, F., and Talwar, K. (2007). Mechanism design via differential privacy. In Proc. of the FOCS'07 (IEEE Computer Society), pp. 94–103. https://doi.org/10.1109/FOCS.2007.41.

Patterns, Volume *2*

Supplemental information

Privacy-preserving data sharing

via probabilistic modeling

Joonas Jälkö, Eemil Lagerspetz, Jari Haukka, Sasu Tarkoma, Antti Honkela, and Samuel Kaski

1 Additional experiment on ARD data

Table S1 shows that using 40 mixture components slightly improves the fit for both male and female cases when compared against both private and non-private results with 10 mixture components.

	Coefficient	Number of cases	Original coef.	$\epsilon = 2.0$	$\epsilon = 4.0$	$\epsilon = \infty$	$\epsilon = \infty, k = 40$
0	OAD only	254	0.657 ± 0.108	0.474 ± 0.209	0.591 ± 0.189	0.887 ± 0.149	0.7 ± 0.121
1	OAD+Insulin	12	0.873 ± 0.304	0.846 ± 0.44	1.074 ± 0.427	1.124 ± 0.366	1.12 ± 0.257
2	Insulin only	117	1.68 ± 0.135	1.085 ± 0.312	1.313 ± 0.293	1.521 ± 0.206	1.587 ± 0.153

	Coefficient	Number of cases	Original coef.	$\epsilon = 2.0$	$\epsilon = 4.0$	$\epsilon = \infty$	$\epsilon = \infty, k = 40$
0	OAD only	1052	0.435 ± 0.049	0.502 ± 0.152	0.538 ± 0.12	0.532 ± 0.089	0.523 ± 0.061
1	OAD+Insulin	66	0.582 ± 0.129	0.816 ± 0.282	0.858 ± 0.234	0.864 ± 0.17	0.757 ± 0.136
2	Insulin only	480	1.209 ± 0.063	1.188 ± 0.205	1.257 ± 0.138	1.262 ± 0.123	1.296 ± 0.082

Table S1: **ARD study**, **ABOVE** : Females, **BELOW** : Males. Increasing the number of mixture components improves the fit. DP and synthetic non-DP ($\epsilon = \infty$) results are average over 100 runs, error denoting the standard deviation. The error in original coefficients shows the standard error for the regression model.
